# Role of environmental factors and microorganisms in determining the fate of polycyclic aromatic hydrocarbons in the marine environment

**DOI:** 10.1093/femsre/fuw031

**Published:** 2016-08-12

**Authors:** Robert Duran, Cristiana Cravo-Laureau

**Affiliations:** Equipe Environnement et Microbiologie, MELODY group, Université de Pau et des Pays de l'Adour, IPREM UMR CNRS 5254, BP 1155, 64013 Pau Cedex, France

**Keywords:** marine microbes, biodegradation, biological pump, *Deepwater Horizon*, microbial assemblages

## Abstract

Polycyclic aromatic hydrocarbons (PAHs) are widespread in marine ecosystems and originate from natural sources and anthropogenic activities. PAHs enter the marine environment in two main ways, corresponding to chronic pollution or acute pollution by oil spills. The global PAH fluxes in marine environments are controlled by the microbial degradation and the biological pump, which plays a role in particle settling and in sequestration through bioaccumulation. Due to their low water solubility and hydrophobic nature, PAHs tightly adhere to sediments leading to accumulation in coastal and deep sediments. Microbial assemblages play an important role in determining the fate of PAHs in water and sediments, supporting the functioning of biogeochemical cycles and the microbial loop. This review summarises the knowledge recently acquired in terms of both chronic and acute PAH pollution. The importance of the microbial ecology in PAH-polluted marine ecosystems is highlighted as well as the importance of gaining further in-depth knowledge of the environmental services provided by microorganisms.

## Abstract (precis)

This review highlights the sources and fate of polycyclic aromatic hydrocarbons (PAHs) in the marine environment with particular emphasis on the microbial ecology and the biological pump controlling global PAH fluxes.

## INTRODUCTION

Polycyclic aromatic hydrocarbons (PAHs) have attracted the interest of many scientists from different disciplines beyond biology, physics and chemistry. They are fascinating compounds due to their universality, their presence in interstellar space (Tielens 2008) and their ubiquitous distribution in earth ecosystems (Vila, Tauler and Grifoll 2015). They have even been hypothesised as crucial elements for the construction of scaffolds at the origin of life (Ruiz-Mirazo, Briones and de la Escosura 2014).

PAHs are natural compounds synthesised by organisms, produced by combustion, and derived from fossil fuels and transformation processes (Neff 2002; Hylland 2006). In the marine environment, they tend to aggregate and to sorb to particulate and organic matter because they are stable hydrophobic planar structures with low solubility and volatility. These physical-chemical properties are related to the presence of at least two benzene rings (Hites, Laflamme and Farrington 1977; Hites, Laflamme and Windsor 1980; Cerniglia 1993). As a consequence, they thus accumulate in the environment (Lu, Zhang and Fang 2011). PAHs tend to be considered persistent molecules, particularly those with a high molecular weight (HMW) (Cerniglia 1992; Wilcock *et al.*^1996^) although recent conceptual models emphasise the importance of environmental parameters in controlling their persistence (Marín-Spiotta *et al.*^2014^). Human activities have considerably increased the level of PAHs in the environment (Finlayson-Pitts and Pitts 1997), particularly in marine ecosystems (Hoffman *et al.*^1984^; Maher and Aislabie 1992), raising serious environmental issues and human health concerns. Some PAHs and their metabolites are considered highly toxic mutagens and carcinogens (Miller and Miller 1981; Dean 1985; White 1986; Mastrangelo, Fadda and Marzia 1996). Their toxicity, which depends on their nature and on environmental factors as well, is increased for PAH mixtures (for a review, see Ball and Truskewycz 2013). Sixteen PAHs have been included in the US Environmental Protection Agency's list of priority pollutants (US-EPA 1984), but this list is now under discussion as some authors propose to include metabolites such as oxy- and nitro-PAHs, which are more toxic than the parent molecules (Andersson and Achten 2015).

As natural compounds, PAHs have been circulating through biogeochemical cycles for millions of years (Henner *et al.*^1997^). Global PAH fluxes in marine environments are controlled by the microbial degradation and the biological pump, which plays a role in sequestration through bioaccumulation and particle settling (Turner 2015). The bioaccumulation of PAHs has been shown in phytoplankton (Binark *et al.*^2000^; Almeda *et al.*^2013^) and marine organisms (Meador *et al.*^1995^). Together with the particulate organic matter and the dissolved organic matter, PAHs are subject to the microbial mineralisation integrating the microbial loop. Thereby, the biological pump and the microbial loop are involved in the fate of PAHs entering the ocean. Jiao *et al.* (2010) proposed the concept of the ‘microbial carbon pump’ that addresses the role of microbial mineralisation of labile dissolved organic matter. The microbial activity also results in the production of recalcitrant dissolved organic matter, which was estimated to represent 155 gigatonnes of carbon with an annual production estimated between 0.008 and 0.023 gigatonnes (Benner and Herndl 2011; Jiao *et al.*^2011^). Gustafsson, Gschwend and Buesseler (1997) have shown that around 90% of PAHs reach deep-sea sediments. This observation has been recently confirmed by Adhikari, Maiti and Overton (2015) in the Gulf of Mexico where they revealed that 3.1%–6.7% of total particulate PAHs were removed daily in the euphotic zone. Microorganisms have developed biodegradation strategies to transform and utilise PAHs as carbon and energy sources. The biodegradation of PAHs by microorganisms has been extensively reviewed for both aerobic (Cerniglia 1992; Kanaly and Harayama 2000; Bamforth and Singleton 2005; Peng *et al.*^2008^; Haritash and Kaushik 2009; Seo, Keum and Li 2009; Lu, Zhang and Fang 2011) and anaerobic (Widdel and Rabus 2001; Bonin *et al.*^2004^; Meckenstock *et al.*^2004^; Foght 2008; Lu, Zhang and Fang 2011; Meckenstock and Mouttaki 2011; Meckenstock *et al.*^2016^; Rabus *et al.*^2016^) processes.

Marine environments cover a large variety of ecosystems as diverse as estuaries, coastal zones, surface and deep oceans in a broad range of latitudes, each of them having specific characteristics. In this review, we summarise the knowledge of PAH biogeochemical cycles in the different marine ecosystems, with a particular focus on the biotic and abiotic factors driving their fate.

## SOURCES OF PAHs IN MARINE ECOSYSTEMS

Over the last decades, PAH contamination has been extensively studied in various marine ecosystems worldwide. Most studies aimed to identify the sources of PAH contamination based on PAH diagnostic ratios. Because PAHs are emitted as mixtures, their profiles are fingerprints reflecting their origin and weathering processes (Manoli, Kouras and Samara 2004). The application of diagnostic ratios and their limitations have been recently reviewed (Tobiszewski and Namieśnik 2012; Stogiannidis and Laane 2015). The diagnostic ratios are usually combined with chemometric approaches, multivariate analyses such as principal component analysis and more recently positive matrix factorization combined with geographic information systems (Mahmoudi *et al.*^2013^), to determine the source apportionment of PAHs (Burns *et al.*^1997^; Tobiszewski and Namieśnik 2012; Lang *et al.*^2013^; Yu *et al.*^2015^), which is pivotal information for risk assessment and management (Tobiszewski and Namieśnik 2012). The main sources of PAHs have been found to be petrogenic and pyrogenic, resulting from anthropogenic activities including direct inputs of petroleum and emissions from the combustion of oil, diesel and biomass (Baek *et al.*^1991^; Oros and Ross 2004; Lima, Farrington and Reddy 2005; Li *et al.*2015b; Wang *et al.*^2015^; Yu *et al.*^2015^). Natural sources from volcanic activities and forest fires have been found to be marginal (Bamforth and Singleton 2005).

**Figure 1.**
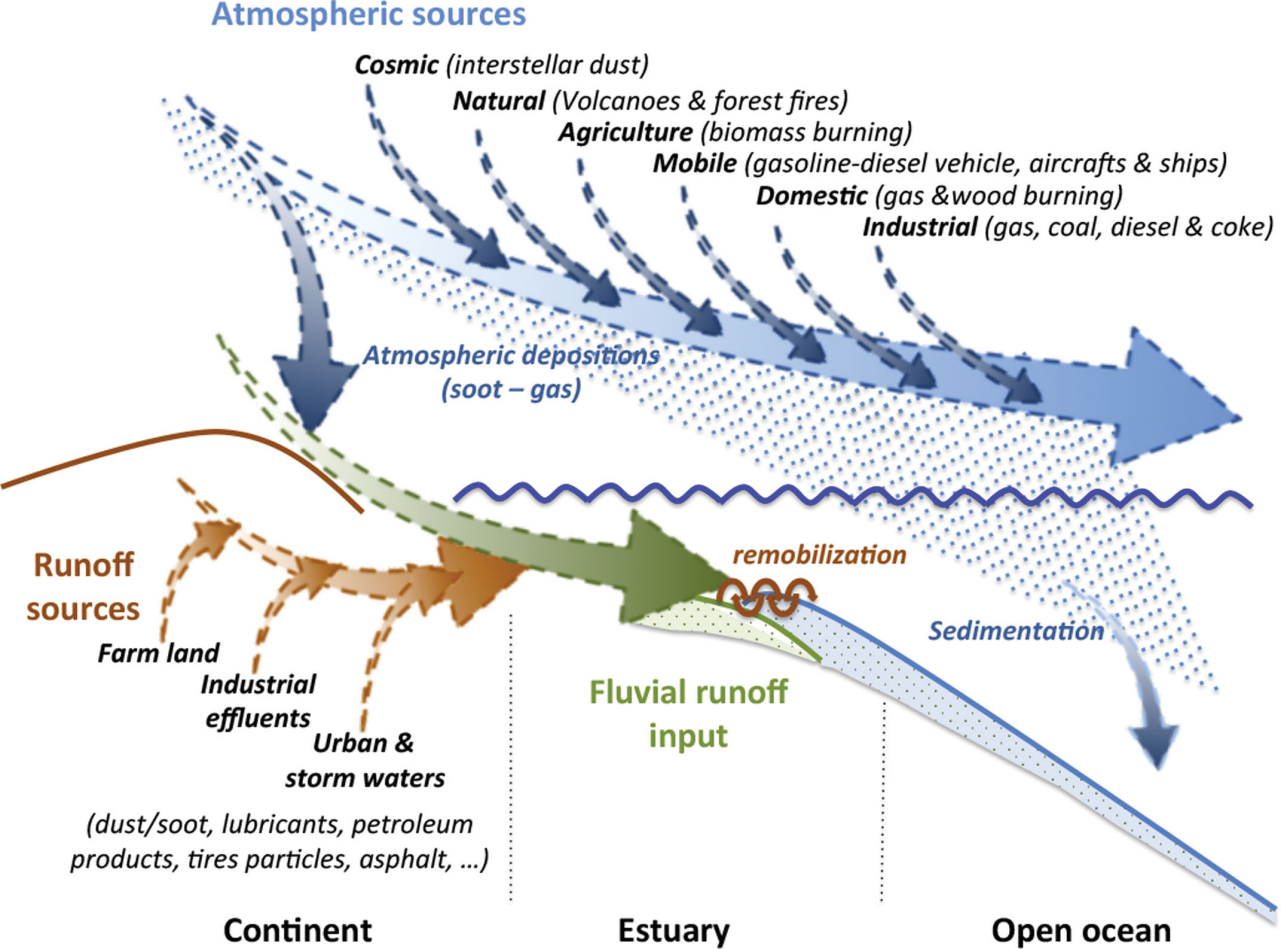
Main sources of pyrogenic PAHs entering the marine environment.

Pyrogenic PAHs, considered as chronic pollution, enter marine ecosystems mainly through fluvial run-off and atmospheric deposition (Fig. 1), whose respective contributions depend on the distance from the point sources (Liu *et al.*^2012^). In distant areas, the relative contribution of water run-off decreases, while that of atmospheric deposition increases (Gogou, Bouloubassi and Stephanou 2000; Chen *et al.*^2006^; Viñas *et al.*^2010^), the latter being almost the principal PAH source in the surface water of open oceans.

The most spectacular quantities of petrogenic PAHs entering marine ecosystems are from oil spills such as the infamous examples of the *Exxon Valdez* in Alaska, the first Gulf War in Kuwait, the *Erika* in France, the *Prestige* in Spain and the more recent accident of the *Deepwater Horizon* in the Gulf of Mexico. Oil spills are considered as acute hydrocarbon pollution, with the aromatic component representing between 3% and 30% according to the type of oil (McGenity 2014). However, such accidental inputs represent only a minor share (less than 10%) of hydrocarbons (including PAHs in varying amounts according to the oil) entering the marine environment. The major sources of PAHs in oceans are from natural oil seeps representing ∼47% of all the crude oil entering the marine environment. The rest is due to transportation and other anthropogenic activities (Judd and Hovland 2007). As oil seeps are generally ancient, they are often considered as natural chronic pollution (Kvenvolden and Cooper 2003; Schroot, Klaver and Schüttenhelm 2005; Judd and Hovland 2007) although petrogenic PAHs are more bioavailable than the pyrogenic PAHs (Baumard, Budzinski and Garrigues 1998). The hydrocarbons released by oil seeps create particular habitats for benthic organisms and exert continuous selection pressure on the microbial community influencing their organization (LaMontagne *et al.*^2004^; Benedetti *et al.*^2014^).

### Coastal ecosystems

Coastlines have diverse ecosystems including estuaries, salt marshes, mangroves, bays, lagoons and coral reefs. These ecosystems provide valuable ecosystemic services and play an important role in the carbon cycle (Bauer *et al.*^2013^), particularly in the mineralisation of organic matter (Burdige 2005; Blair and Aller 2012). With ∼4 billion people living within 60 km of the world's coasts (Kennish 2002), these marine coastal ecosystems are strongly impacted by human activities (Halpern *et al.*^2008^). PAHs threaten sensitive coastal ecosystems (McGenity 2014) including mangroves (Ke *et al.*^2005^; Mille *et al.*^2006^; Brito *et al.*^2009^; Bayen 2012), salt marshes (Duran and Goñi Urriza 2010; Coulon *et al.*^2012^; Chronopoulou *et al.*^2013^), coral reefs (Burns 2014; Ko, Chang and Cheng 2014; Guigue *et al.*^2015^) and even Arctic coasts with increasing traffic due to the diminution of the polar ice cap (Jörundsdóttir *et al.*^2014^).

The most urbanised coastal areas, such as estuaries, harbours and marinas, constitute hot spots of multicontaminants that may lead to the ‘coastal pollution and contamination syndrome’, whose consideration as a ‘tipping element’ (Schellnhuber 2009) in the global Earth system is discussed by Newton, Carruthers and Icely (2012). In particular, PAHs are a major concern in estuarine sediments (Christiansen *et al.*^2009^) with concentration levels often exceeding 100 ng g^−1^ dry weight sediment, above the lowest pollution level as defined by Baumard, Budzinski and Garrigues (1998). This value is an order of magnitude lower than the ERL (effects range-low) concentration for causing harmful effects in biota (4022 ppb per dry weight sediment, Long *et al.*^1995^). Nonetheless, many sites around the world show risks associated with PAH contamination in sediments (Burgess *et al.*^2013^) presenting PAH concentration levels above the sediment quality guideline (SQG) values proposed for total PAHs, such as the ERL and the TEL (threshold effects level) for causing occasional adverse effects (1684 ppb per dry weight sediment, Macdonald *et al.*^1996^). The different SQG approaches for sediment risk assessment have been recently reviewed by Burgess *et al.* (2013). However, the dilution of pollution in the vast expanse of the ocean reduces the environmental risks. For example, the PAH content during the *Deepwater Horizon* catastrophe decreased with distance and time in the water column (Boehm, Murray and Cook 2016) and sediments (Adhikari *et al.*^2016^). A clear decrease in PAH levels in seawater was observed after the well was capped (Wade *et al.*^2016^). A major PAH depletion (over 70%) mainly due to abiotic processes was observed in floating and stranded oils (Stout *et al.*^2016^). However, the PAH content remained high in some areas, particularly affected by the oil spill such as Louisiana coastal wetlands (Turner *et al.*^2014^). Nevertheless, most of the sediments were considered unlikely to cause toxic effects since the PAH contents were below the ERL (Wang *et al.*^2014^).

Because estuaries are ecosystems with complex hydrodynamics and the human activities carried out there are so diverse, each estuary may represent a case study with its own specificity. The estimation of PAH fluxes from the different sources is a valuable element in characterising the PAH input mechanism (Liu *et al.*^2012^) and determining the river contribution at a global scale as well (Wang *et al.*^2007^). For example, in highly urbanised areas, the urban and storm waters are the most significant PAH inputs, as shown for the Seine Estuary in France (Motelay-Massei *et al.*^2007^), Tokyo Bay in Japan (Pan *et al.*^2014^) and the estuaries of Massachusetts in the USA (Menzie *et al.*^2002^). In industrialised areas, the main sources of PAHs are mainly from industrial wastewaters as reported for the Pearl River Delta in China (Mai *et al.*^2002^; Wang *et al.*^2007^), or coal and biomass combustion deposits as determined for the estuaries of the Linhong River (Zhang, Zhang and Zhang 2013) and the Yangtze River (Guo *et al.*^2007^) in China, or petroleum combustion deposits in Malaysian estuaries (Keshavarzifard *et al.*^2015^), the San Francisco Estuary in the USA (Oros and Ross 2004) and the Mersey Estuary in the UK (Vane, Harrison and Kim 2007). The main contribution of direct petrogenic sources has been identified mainly in harbours as evidenced for the Yangshan Port in China (Li *et al.*2015a), the Imam Khomeini Port in Iran (Abdollahi *et al.*^2013^) and the Olbia harbour in Italy (De Luca *et al.*^2005^).

### Open-Sea ecosystems

Oceans cover 70% of the Earth's surface and major gas exchanges occur at the ocean–atmosphere interface (Nizzetto *et al.*^2008^). Oceanic water bodies are thus the final sink of atmospheric pollutants that are transferred to deep waters and finally trapped into sediments (Dachs *et al.*^2002^). The pyrogenic PAH composition in the atmosphere is dependent on their sources (Tsapakis *et al.*^2006^; González-Gaya *et al.*^2014^). Atmospheric PAH fluxes are affected by physical-chemical properties and environmental variables, such as wind speed, collisions with the marine surface, seawater surface tension and hydrophobicity (González-Gaya *et al.*^2014^). PAHs are characterized by short atmospheric half-lives (Mackay, Shiu and Ma 1997) due to their affinity to soot carbon (Dachs, Eisenreich and Hoff 2000) that facilitates their deposition in seawater. Furthermore, higher primary production enhances air–water exchange and vertical PAH sinking fluxes (Dachs, Eisenreich and Hoff 2000; Dachs *et al.*^2002^). Phytoplankton influences air–water exchanges both directly by PAH sorption and indirectly. Growth rates induce the depletion of the dissolved phase concentration causing an air–water phase disequilibrium (Dachs *et al.*^1999^).

The petrogenic PAHs from natural oil seeps are the other source of PAHs in open seas. The petrogenic/pyrogenic dual composition of PAHs is illustrated in the Norwegian Sea, where Boitsov *et al.* (2013) demonstrated that the PAH composition determined in the seabed (non-pyrogenic PAHs) was different to that found in coastal areas (pyrogenic PAHs). The PAH composition in open sea surface sediments was characterized by a petrogenic/pyrogenic PAH mixture (Boitsov *et al.*^2013^; Buhl-Mortensen *et al.*^2015^), but presenting variations at some locations. Similarly, in the Arctic Ocean different PAH sources have been identified including long-range atmospheric transport (Friedman and Selin 2012) and petrogenic origin from natural oil seeps (Foster *et al.*^2015^). Conversely, in the Mediterranean Sea, the atmospheric deposition dominated by pyrogenic PAHs was found to be predominant in the open sea (Lipiatou and Saliot 1991; Tsapakis *et al.*^2006^). Additionally, Tsapakis *et al.* (2006) demonstrated that low molecular weight (LMW) PAHs enter the seawater via diffusive gas exchanges and HMW PAHs through dry and wet deposition. HMW PAHs are generally associated with soot particles and black carbon (BC) (Shrestha, Traina and Swanston 2010). In a north/south transect in the open Atlantic Ocean, Nizzetto *et al.* (2008) showed that atmospheric deposition/air–water exchanges were the principal processes by which PAHs enter the Atlantic seawater. However, the PAH concentrations were found to be higher in the North Atlantic waters than in the South Atlantic waters, showing the impact of the industrialisation.

## ABIOTIC FACTORS DETERMINING THE FATE OF PAHs IN MARINE ECOSYSTEMS

**Figure 2.**
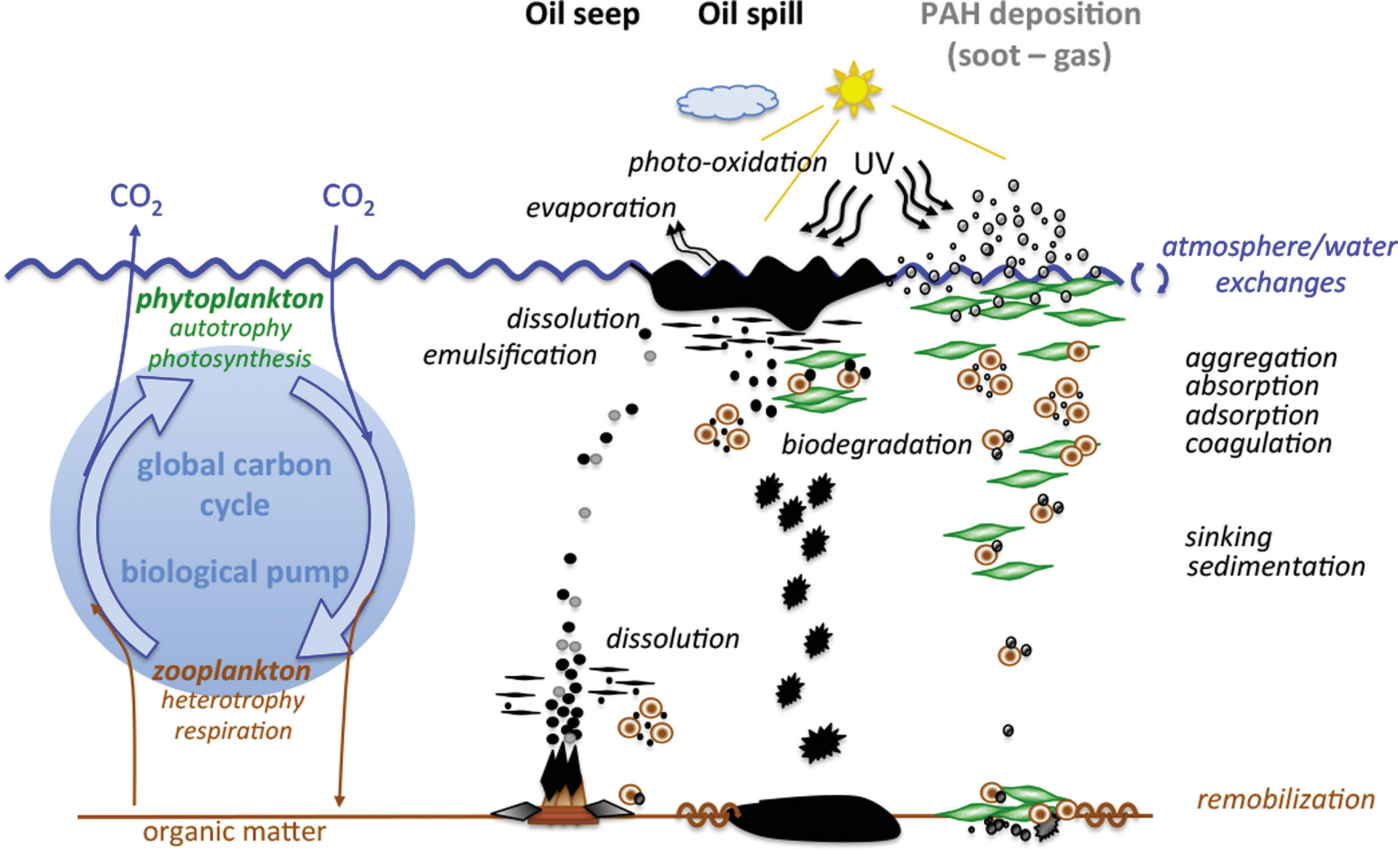
Main processes determining the fate of oil and PAHs in the marine environment. Atmospheric PAH deposition on surface waters, corresponding to most cases of chronic pollution and petrogenic PAHs entering by oil spills, corresponding to acute pollution, undergo similar global abiotic and biotic processes. PAHs from natural oil seeps travel through the water column to the surface and finally follow the same processes as the previously described PAHs. The microbial biodegradation and the biological pump control PAH fluxes. The latter plays a crucial role in atmospheric PAH sequestration, bioaccumulation and sedimentation.

The way in which PAHs enter marine ecosystems is crucial in determining their bioavailability and fate. Petrogenic PAHs are more bioavailable than those of pyrogenic origin, because the latter are tightly bound to particles (Wade, Sweet and Klein 2008), and therefore more strongly associated to sediments (Perra *et al.*^2009^). Indeed, the degradation of fossil petrogenic PAHs was found to be higher than that of pyrogenic PAHs during transport through the water column in the Mediterranean Sea (Dachs *et al.*^1997^). Although similar general processes are involved, we make the distinction between chronic and acute PAH pollution. We thus describe specific characteristics of the abiotic fate for pyrogenic PAHs entering the marine environment by deposition on surface waters, typical of most cases of chronic pollution and for petrogenic PAHs entering the marine environment due to oil spills, which correspond to acute pollution (Fig. 2).

### Chronic PAH pollution—fate of water-surface-deposited PAHs

The incomplete burning of biomass and fossil fuel produces BC, soot and char particles that are considered as long-term persistent in the environment because they are refractory to biological and chemical decomposition processes (Middelburg, Nieuwenhuize and Van Breugel 1999). BC is now considered a major global pollutant playing a primary role in climate change by affecting the radiation equilibrium and modifying surface albedo (for a review, see Novakov and Rosen 2013). BC is also considered as an important component of the carbon cycle (Lohmann *et al.*^2009^), being a recognised carbon sink sequestered over centuries (Bird *et al.*^2015^). The main processes involved in the formation of PAHs during BC production have been described (for a review, see Richter and Howard 2000; Lima, Farrington and Reddy 2005). PAH levels and composition in BC are dependent on the type of fuel burned and the burning conditions (Jenkins *et al.*^1996^; Schauer *et al.*^2002^; Simoneit 2002). Flores-Cervantes, Reddy and Gschwend (2009b) defined three main BC types: (i) thermally recalcitrant/highly sorptive, (ii) thermally labile/highly sorptive and (iii) thermally recalcitrant/not highly sorptive. In addition to the PAHs bound by occlusion into BC pores during soot formation, BC also binds organic pollutants, particularly PAHs through surface sorption processes (Shrestha, Traina and Swanston 2010). BC is thus an important PAH transporter both in the atmosphere and in the seawater column (Lohmann, Macfarlane and Gschwend 2005; Flores-Cervantes *et al.*2009a), and BC is acknowledged to play a major role in controlling the fate and bioavailability of hydrophobic organic pollutants (Shrestha, Traina and Swanston 2010). Pyrogenic PAHs emitted as soot particles and gas enter the water column by direct or rainfall depositions of soot particles (i.e. BC—HMW PAHs) and by gas exchange at the water/atmosphere interface for volatile (i.e. LMW) PAHs (Oros and Ross 2004). The annual BC production has been estimated to be between 16 and 275 Tg BC yr^−1^ (Flores-Cervantes *et al.*2009a). Jurado *et al.* (2008) estimated the dry deposition of total organic carbon to be 11 Tg C yr^−1^ and the wet deposition of particle and gaseous total organic carbon to be 47 and 187 Tg C yr^−1^, respectively. The global ocean BC uptake was estimated to be 2 and 10 Tg C yr^−1^ by dry and wet deposition, respectively (Jurado *et al.*^2008^; Shrestha, Traina and Swanston 2010).

Once in the water column, due to their low water solubility and hydrophobic nature, PAHs sorb rapidly to organic and inorganic particulate material sinking to the bottom and accumulate in sediment (Tolosa *et al.*^2004^). Deep-sea sediments and the open ocean are thus considered as the major sink for PAHs (Adhikari, Maiti and Overton 2015). Although the dynamics of PAHs in marine environments depend on diverse mechanisms including photo-oxidation, evaporation, dispersion and biodegradation (Berrojalbiz *et al.*^2009^), the vertical sinking of PAH particles is an important process in determining their fate, as summarised by Adhikari, Maiti and Overton (2015). A recent study demonstrated that 3.1%–6.7% of total particulate PAHs in the water column were lost daily via vertical sinking in the northern Gulf of Mexico (Adhikari, Maiti and Overton 2015). It has been shown that phytoplankton plays an important role in pollutant uptake (including both adsorption and absorption processes) and vertical transport (Dachs *et al.*^2002^). However, dissolved BC in marine-dissolved organic carbon constitutes an intermediate BC pool prior to sediment deposition (Masiello and Druffel 1998; Ziolkowski and Druffel 2010). BC has been estimated to contribute up to 5% of total dissolved organic carbon (DOC) (Stubbins, Niggemann and Dittmar 2012; Coppola, Walker and Druffel 2015; Coppola and Druffel 2016). The concentration of particulate PAHs in the water column is expected to decrease with depth because PAHs are desorbed and degraded during vertical transport (Lipiatou and Saliot 1991; Dachs *et al.*^1997^; Berrojalbiz *et al.*^2009^). Dachs *et al.* (1997) described the depth-depletion distribution of PAHs associated with suspended particulate matter, estimated from the concentration found in vertical profiles, proposing a budget of PAHs in the western Mediterranean seawater. Photo-oxidation degradation processes have been demonstrated to remove aromatic BC producing less condensed dissolved BC (Stubbins, Niggemann and Dittmar 2012; Wagner and Jaffé 2015). It is likely that the BC cycling rates throughout the surface photic zone determine the persistence of dissolved BC in the oceans (Stubbins, Niggemann and Dittmar 2012). The remineralisation of particulate organic carbon containing attached PAHs results in PAH release into the water column in colloidal and/or some soluble forms (Shrestha, Traina and Swanston 2010). Abiotic and biotic PAH degradation may also occur in a higher proportion for LMW PAHs, but degradation processes may be limited (Santín *et al.*^2016^). The transport to deep layers is determined by the biological characteristics of the ecosystem. The organic matter flux in deeper layers has been observed to be higher in eutrophic sites than in oligotrophic areas (Baines, Pace and Karl 1994).

### Acute pollution—fate of PAHs from oil spills

Crude oil hydrocarbons and gas hydrocarbons released from the deep sea, as observed for the *Deepwater Horizon* oil spill or natural oil seeps, partially dissolve in the water column, introducing PAHs and other aromatic compounds into the seawater (Reddy *et al.*^2012^). Diercks *et al.* (2010) have reported the presence of PAHs at concentrations reaching 189 μg/L in the Gulf of Mexico after the *Deepwater Horizon* oil spill where DOC was observed at 6 mg C/L (Zhou *et al.*^2013^). The baseline values of DOC in the northern Gulf of Mexico were considerably lower (usually <1 mg C/L) before the *Deepwater Horizon* oil spill (Guo, Coleman and Santschi 1994; Guo, Santschi and Warnken 1995), highlighting the influence of released oil in DOC concentration. The *Deepwater Horizon* oil spill was characterized by the formation of a hydrocarbon plume at 1000–1300 m depth, probably as a consequence of high-pressure oil released into cold water (Socolofsky, Adams and Sherwood 2011). As described for tanker accidents, such oil spills also lead to crude oil slicks at the surface that undergo weathering processes, dispersing oil droplets through the water column, forming ‘chocolate mousse’ (water-in-oil emulsion), modifying their chemical composition and sinking to the sediment adsorbed to particles (McGenity *et al.*^2012^). The involvement of phytoplankton and marine snow in hydrocarbon sinking was demonstrated during the *Deepwater Horizon* oil spill (Passow *et al.*^2012^; Joye, Teske and Kostka 2014; Passow *et al.*^2014^). The main modifications include the evaporation of LMW hydrocarbons (McGenity *et al.*^2012^), the production of oxygenated PAHs (oxy-PAHs) via biological, chemical and photo-oxidation reactions (Lundstedt *et al.*^2007^) and the formation of tar balls and oil–mineral aggregates (Christiansen *et al.*^2009^) by aggregation and adsorption with minerals (Kiruri, Dellinger and Lomnicki 2013). Tar balls were found to have accumulated and be persistent in coastal sediments after the *Prestige* (Serrano *et al.*^2006^; Acosta-González *et al.*^2015^) and *Deepwater Horizon* oil spills (Beazley *et al.*^2012^; Kiruri, Dellinger and Lomnicki 2013). The production of oxy-PAHs by photo-oxidation reactions from both petrogenic and pyrogenic PAHs increases their solubility but can also result in ‘dead-end products’ resistant to biodegradation that accumulate in sediments threatening human health and the environment (Lundstedt *et al.*^2007^). Although the potential toxic effect of oxy-PAHs depends on the compounds, oxy-PAHs are often more toxic than the parent PAH molecules. The toxic effect of oxy-PAHs has been demonstrated on a large variety of aquatic and terrestrial organisms including microorganisms, algae, plants and mammalian cells (for a review, see Lundstedt *et al.*^2007^). The toxic effect of oxy-PAHs involves oxidative stress and endocrine-disruption mechanisms. They have also been found to be mutagenic and genotoxic through the formation of DNA adducts (for a review, see Bolton *et al.*^2000^; Lundstedt *et al.*^2007^).

Such oxy-PAH compounds have been found in diverse PAH-contaminated environments (Lundstedt *et al.*^2007^; Layshock, Wilson and Anderson 2010) including *Deepwater Horizon* oil spill impacted sediments (Aeppli *et al.*^2012^; Forsberg *et al.*^2014^). The oxy-PAH level has been estimated at up to 12% of total PAHs in marine sediments (Layshock, Wilson and Anderson 2010). The oxygenated hydrocarbon content was found to be between 50% and 65% in weathered *Deepwater Horizon* oil slicks and sand patties (Aeppli *et al.*^2012^). Oxy-PAHs have been found together with other products from photoreactions such as hydroxy-PAHs and nitro-PAHs in atmospheric depositions (Barrado *et al.*^2013^; Sippula *et al.*^2014^) and oil spills (Kleindienst, Paul and Joye 2015).

### Fate of PAHs in sediments

Whatever the origin of contamination, PAHs finally reach the sediments, which represent the main sink for PAHs and constitute PAH reservoirs. Analyses of PAHs in sediment cores are useful chronometers for retracing the history of the pollution (Zhang, Zhang and Zhang 2013). Several studies have demonstrated the industrial PAH input with increasing levels until the 1950s and, following shifts in the use of coal/oil/gas energy sources, have highlighted a continuous decrease in PAH levels initiated in the 1960s with the first countermeasures to limit emissions (Lima, Eglinton and Reddy 2003; Louchouarn *et al.*^2012^).

Sediments, particularly coastal sediments, have been demonstrated as PAH sources for the water column (King, Readman and Zhou 2004; Sabin *et al.*^2010^). The remobilisation of PAHs, experimentally demonstrated in a sediment resuspension simulator (Feng *et al.*^2007^), has been shown to be a highly complex process in the natural environment, depending on several factors including the physical-chemical properties of PAHs and the presence of other pollutants, sediment composition, environmental conditions and hydrologic dynamics (King, Readman and Zhou 2004). It takes place when sediments are disturbed, by either mechanical (shipping, currents, storms, waves and tide) or biological processes (bioturbation), removed or relocated by dredging (for a review, see Roberts 2012). Remobilisation and resuspension processes affect benthic communities (Roberts 2012), drive particular microbial assemblages and activities by providing oxygen deeper in sediments and bioavailable PAHs amenable to biodegradation (LeBlanc *et al.*^2006^). PAH biodegradation by bacteria, fungi and algae has been shown as the main mechanism removing PAHs from marine sediments (McGenity *et al.*^2012^). PAH biodegradation has been shown even in the absence of molecular oxygen by anaerobes (for a review, see Kimes *et al.*^2014^; McGenity 2014; Meckenstock *et al.*^2016^). However, the degradation rates are low for PAHs buried in sediments, as observed after the *Deepwater Horizon* oil spill that resulted in PAH accumulation in deep sediments (Turner *et al.*^2014^; Chanton *et al.*^2015^; Adhikari *et al.*^2016^). Laboratory experiments have demonstrated that PAH partitioning towards the sorbed phase is controlled by sediment grain size, salinity and temperature (Frapiccini and Marini 2015).

## MICROBIAL PAH DEGRADATION

**Figure 3.**
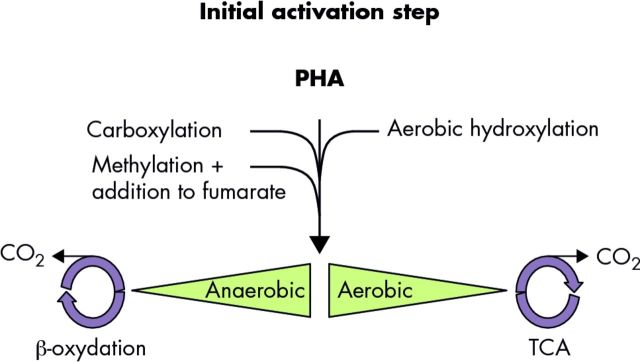
Main activation processes in PAH biodegradation. The first step in the aerobic PAH catabolic pathways involves RHD. For the anaerobic PAH catabolic pathways, the activation steps presumably involve methyl-transferases for methylation, succinate synthases (glycyl radical enzymes) for addition to fumarate and carboxylases for carboxylation. The metabolic pathways have been largely reviewed (Lu, Zhang and Fang 2011; Meckenstock and Mouttaki 2011; Heider and Schühle 2013).

Microorganisms have developed diverse strategies to activate PAH molecules, the initial steps in biodegradation pathways, even in the absence of molecular oxygen (Fig. 3). These mechanisms have been nicely reviewed recently (Kimes *et al.*^2014^; McGenity 2014). Under aerobic conditions, the first activation step involves direct incorporation of oxygen by di- and mono-oxygenases. The key enzymes are ring-hydroxylating dioxygenases (RHDs) belonging to the Rieske-type non-heme iron oxygenase family (Pieper, Martins Dos Santos and Golyshin 2004). Among the PAH-specific RHD, phylogenetic analyses discriminate between the PAH RHDs from Gram-negative bacteria and those from Gram-positive bacteria (Kweon *et al.*^2008^). The main Gram-negative representative PAH RHD genes are the *nagAc* gene from *Ralstonia* sp. U2 and the *nahAc* gene from *Pseudomonas* sp. 9816–4, while the *nidA* gene from *Mycobacterium* is representative of the PAH RHD genes from Gram-positive bacteria (Wu *et al.*^2014^). The PAH RHD incorporates two oxygen molecules into an aromatic ring forming a *cis*-dihydrodiol, which is converted into a catechol by a dehydrogenase. Then, a catechol dioxygenase performs the aromatic ring fission by *ortho-* or *meta-*cleavage producing aliphatic products that enter the central metabolism via the tricarboxylic acid cycle (Cerniglia 1992). Monooxygenases, such as the cytochrome P450 for eukaryotic microorganisms, are involved in detoxifying pathways rather than PAH assimilation processes (Doyle *et al.*^2008^). They are enzymes showing a wide substrate spectrum that can introduce one oxygen atom into the aromatic ring, resulting in an arene oxide intermediate subsequently transformed to either a dihydrodiol by an epoxide hydrolase or a phenol through non-enzymatic rearrangement (Cerniglia 1992; Haritash and Kaushik 2009).

Under anaerobic conditions two main mechanisms have so far been described for PAH molecule activation (Fig. 3). Anaerobic activation mechanisms include direct carboxylation or methylation followed by addition to fumarate (Heider and Schühle 2013; Meckenstock *et al.*^2016^; Rabus *et al.*^2016^). The enzymes involved in the anaerobic pathways have been described in detail (for a review, see Meckenstock *et al.*^2016^). For the carboxylation route, a carboxylase belonging to the UbiD-like proteins family is proposed for the carboxylation of the aromatic ring (Rabus *et al.*^2016^). For the methylation route, it is proposed to proceed via a methyl-transferase that methylates the aromatic ring (Safinowski and Meckenstock 2006) and then a naphthyl-2-methyl-succinate synthase, a glycyl-radical-containing enzyme, catalyses the addition to fumarate (Meckenstock and Mouttaki 2011). In both cases, the degradation pathway further proceeds via β-oxidation after activation with coenzyme A (Meckenstock *et al.*^2016^).

## MICROBIAL ECOLOGY OF PAH-POLLUTED MARINE ECOSYSTEMS

Although PAH microbial degradation mechanisms are well known, the ecology of microbial communities facing the presence of PAHs in marine environments requires further elucidation due to the myriad of factors that can contribute to and/or affect microbial community structure in different polluted environments (Cravo-Laureau and Duran 2014; Duran *et al.*2015c). The particle grain size and dissolved oxygen were revealed as the main drivers for bacterial diversity and abundance in estuarine sediments (Wang *et al.*^2013^) and deep ocean waters (Salazar *et al.*^2015^). However, specific microbial assemblages and hydrocarbonoclastic bacterial strains have been described for chronically contaminated coastal sediments (Paissé *et al.*^2008^; Ben Said *et al.*^2010^; Duran *et al.*2015a; Misson *et al.*^2016^) and for waters and sediments contaminated by oil spills (Bordenave *et al.*2004a,b; Stauffert *et al.*^2013^; Kimes *et al.*^2014^; Acosta-González *et al.*^2015^; Stauffert, Cravo-Laureau and Duran 2015a). Particular hydrocarbon degrading bacterial strains have been found in deep ocean waters such as members related to the *Maribaculum*, *Novosphingobium*, *Oceanibaculum*, *Parvibaculum*, *Roseovarius* and *Stappia* genera as well as in deep sediments including members related to the *Oceanicola*, *Parvibaculum*, *Nitratireductor*, *Celeribacter* and *Bowmanella* genera (for a review, see Louvado *et al.*^2015^). The genomes of the PAH-degrading bacterial isolates from deep-sea sediments carry potential new PAH RHD genes with low sequence similarity to known genes involved in the initial activation step (Louvado *et al.*^2015^). Such observation suggested that the genetic potential for PAH degradation may be greater than previously recognised. Some isolates from deep-sea sediments also harbour known genes probably acquired via horizontal gene transfer (Cao *et al.*^2015^).

The occurrence of hydrocarbon-degrading bacteria in PAH-polluted sites, their role in PAH biodegradation and their ecological significance have been largely discussed (for a review, see McGenity *et al.*^2012^; Kimes *et al.*^2014^; McGenity 2014; Louvado *et al.*^2015^). However, despite the information available, the generalisation of microbial patterns in PAH-contaminated sites is made difficult because of the wide variety of marine ecosystems, the origin of contamination and the environmental conditions (Nogales *et al.*^2011^). A recent large-scale study in the Mediterranean Sea revealed that the microbial communities in pristine sites responded in a different way to those inhabiting chronically polluted sites (Bargiela *et al.*^2015^). The latter showed a faster degradation response when an accidental oil spill occurred. Bargiela *et al.* (2015), by comparing metagenomic-based pollutant-degrading networks of chronically polluted sites in the Mediterranean Sea with those of the *Deepwater Horizon* oil spill, demonstrated that chronic pollution promotes the diversification of PAH catabolic capacities. The study also revealed a correlation between temperature and microbial diversity, with low temperature increasing bacterial richness while decreasing catabolic diversity (Bargiela *et al.*^2015^).

### Oil spill microbial ecology

The microbial ecology of oil spills has been studied extensively (for a review, see McGenity *et al.*^2012^; Cravo-Laureau and Duran 2014; McGenity 2014; Acosta-González and Marqués 2016). Here we focus mainly on the most recent oil spill, the *Deepwater Horizon* oil spill, which largely increased the input of PAHs in the Gulf of Mexico (Reddy *et al.*^2012^), although the PAH content of the Macondo MC252 crude oil was estimated between 1.1% and 4% (Reddy *et al.*^2012^; Daling *et al.*^2014^; Yin *et al.*^2015^). It is important to note that the Gulf of Mexico is a chronically polluted environment with numerous natural oil seeps that contribute more than 400 000 barrels of oil per year (Atlas and Hazen 2011; Farrington 2013). The *Deepwater Horizon* disaster was thus a major oil spill in a chronically polluted environment providing an input of bioavailable hydrocarbons and PAHs to adapted microbial communities (Atlas and Hazen 2011; Atlas *et al.*^2015^). The *Deepwater Horizon* oil spill was characterized by the use of dispersants as a remediation strategy. The consequences and impact of dispersant application have been recently reviewed (Kleindienst, Paul and Joye 2015). The role of dispersants is to allow the dispersion of insoluble hydrocarbon compounds (Prince *et al.*^2013^). Contrasted effects of dispersants on hydrocarbon microbial degradation have been recently reported. Several studies have demonstrated the efficiency of dispersants in hydrocarbon dilution promoting their microbial degradation (Lee *et al.*^2013^; Prince *et al.*^2013^; Prince 2015), while recent studies have revealed that dispersants can exert a negative effect on microbial hydrocarbon degradation rates (Kleindienst *et al.*^2015^) and affect the macrofaunal activity (Cuny *et al.*^2015^). Thus, these observations point out the necessity to further understand the effect of dispersant on biodegradation, preferentially in realistic environmentally relevant conditions because most studies conducted so far under laboratory conditions do not reflect the *in situ* conditions. Studies taking into account the type and amount of dispersant as well as considering the whole complex microbial community in a holistic point of view are particularly needed for a fully conscious use of dispersants (Kleindienst, Paul and Joye 2015).

The *Deepwater Horizon* disaster provided a perfect ecosystem model for studies on the fate of oil in the marine environment. A large literature is now available, particularly on microbial ecology aspects, confirming the important role of microorganisms in determining the fate of spilled oil. Among this large literature, three reviews (Joye, Teske and Kostka 2014; Kimes *et al.*^2014^; King *et al.*^2015^) have been recently published addressing the microbial response to the *Deepwater Horizon* oil spill and the underlying ecological rules.

In this ecosystem, although environmental conditions such as nutrients or temperature could limit microbial activities, PAH biodegradation by microorganisms has been evidenced. Moreover, an ecological succession of microorganisms during the hydrocarbon degradation has been highlighted (Joye, Teske and Kostka 2014). Such ecological succession has been described as the main rule of microbial oil response (Head, Jones and Roling 2006) and observed in other oil spills such as the *Prestige* oil spill, mainly composed of PAHs (Acosta-González *et al.*^2015^). Similar trends have also been observed with experimental ecology approaches simulating oil spill and mimicking environmental conditions (Cravo-Laureau and Duran 2014) showing a modification in benthic microbial communities during experimental exposure (Stauffert *et al.*^2013^, ^2014^; Stauffert, Cravo-Laureau and Duran 2015a,b). All these observations highlight the functional redundancy in hydrocarbon degradation characterized by different compositions of microbial communities, although the efficacy in hydrocarbon degradation and removal remains similar (Head, Jones and Roling 2006; Stauffert *et al.*^2013^; Cravo-Laureau and Duran 2014).

It has been suggested that Gulf of Mexico deep-sea microbial communities were adapted to oil because they were exposed to natural oil seeps (Joye, Teske and Kostka 2014). The link between microbial degradation efficiency and pollution history has been largely discussed (Head, Jones and Roling 2006; Bordenave *et al.*^2007^) and remains under debate (Sauret *et al.*^2012^). The idea that microbial communities with pollution history respond faster than a pristine microbial community is supported by the fact that the response of the Gulf of Mexico bacterial communities to the presence of the Macondo *Deepwater Horizon* oil was extremely fast, characterized by the enrichment of the deep-sea oil plume (1000–1300 m depth) by indigenous oil-degrading bacteria (Hazen *et al.*^2010^) as well as by bacterial blooms and marine snow phenomena (Joye, Teske and Kostka 2014). The marine oil snow, also referred to as a ‘dirty blizzard’, was characterized by ecological succession, with the presence and dominance of members related to *Cycloclasticus* genus associated with the presence of PAHs (Joye, Teske and Kostka 2014). Biological snow production by the association of bacterial and/or algal mucus, with phytoplankton and zooplankton faecal debris and feeding, was studied during the *Deepwater Horizon* oil spill (Passow *et al.*^2012^; Vonk, Hollander and Murk 2015). The studies demonstrated that a significant proportion of the spilled oil was carried to the seafloor during snow sinking. During sedimentation, biodegradation of the most labile fraction (e.g. LMW PAHs) was demonstrated (Ziervogel, Joye and Arnosti 2016; Joye, Teske and Kostka 2014). Once at the sediment surface under oxygen-depleted conditions, anaerobic bacteria, such as sulphate-reducing Deltaproteobacteria, replaced the aerobic bacteria (Joye, Teske and Kostka 2014). In a recent review, Vonk, Hollander and Murk (2015) investigated whether marine oil snow occurred in other large oil spills by meta-analysis, and concluded that marine oil snow and related benthic contamination may be widespread phenomena in response to pollution.

Rodriguez-R *et al.* (2015) demonstrated that, a year after the catastrophe, the ecological succession resulted in the general recovery of diversity with the reoccurrence of specialised and sensitive microbial populations. The resistance and resilience of microbial communities are important issues in microbial ecology (Allison and Martiny 2008; Nogales *et al.*^2011^), which was essentially so far described through oil exposure in experimental microcosms oil (Bordenave *et al.*^2007^). The *Deepwater Horizon* disaster at the Gulf of Mexico is a perfect *in situ* case study to follow microbial communities behaviour. PAH biodegradation was demonstrated, for example by metatranscriptomic approaches, revealing the expression of PAH degradation genes in oiled sediments (Lamendella *et al.*^2014^), or by culture-dependent approaches using SIP (Gutierrez *et al.*^2013^). Considering the microbial community related to PAH biodegradation, microorganisms belonging to the genera *Cycloclasticus, Colwellia* and *Alteromonas* have been described as abundant in the water column and surface water (Valentine *et al.*^2010^; Gutierrez *et al.*^2013^), while in deep-sea sediments a high level of sulphate-reducing microorganisms have been observed (Orcutt *et al.*^2010^; Kimes *et al.*^2014^). Associating cultivation-based and cultivation-independent molecular analyses of bacterial communities, Kostka *et al.* (2011) proposed to use members of Alphaproteobacteria (*Labrenzia* and *Rhodobacteraceae*) and Gram-positive groups (*Bacillus* and *Microbacterium*) as sentinels for PAH biodegradation in Gulf beach sands. In coastal wetlands, polluted by weathered oil containing complex PAHs (Beazley *et al.*^2012^), oil degradation was enhanced by the increasing oxygenation of sediments by bioturbation or by the presence of rhizosphere marsh vegetation that also releases exudates. The microbial community was dominated by phyla containing previously described hydrocarbon-degrading bacteria (Proteobacteria, Bacteroidetes and Actinobacteria) and functional genes involved in PAH degradation were detected (Beazley *et al.*^2012^).

As evidenced by the available literature, considerable information has been obtained on bacterial communities and PAH biodegradation. Considering Archaea, their involvement in PAH degradation has been demonstrated particularly by halophilic microorganisms (McGenity 2010; Bonfá *et al.*^2011^; Andrei, Banciu and Oren 2012) as shown by Bertrand *et al.* (1990), who isolated the first halophilic strain able to degrade PAHs (to a lesser extent than aliphatic hydrocarbons) from a salt marsh, a marine coastal ecosystem where Archaea represent an important component (Sanni, Coulon and McGenity 2015). PAH biodegradation has also been observed under methanogenic conditions (Chang, Um and Holoman 2006). The impact of PAH pollution on archaeal communities has been indirectly addressed, by monitoring the effect of oil pollution. Only a few studies have been reported on this, with contrasting results (Röling *et al.*^2004^; Taketani *et al.*^2010^; Stauffert *et al.*^2014^; Sanni, Coulon and McGenity 2015). Modifications in the composition of archaeal communities have been described during oil spills (Newell *et al.*^2014^; Stauffert *et al.*^2014^), the nitrification community being particularly affected by hydrocarbon toxicity (Urakawa *et al.*^2012^). In contrast, no effect has been observed in the archaeal ammonia oxidizer community (Rivers *et al.*^2013^) and more recently, Yergeau *et al.* (2015) reported this community to be dominant in the polluted area they studied.

Although fungi have a high PAH degradation potential (Harms, Schlosser and Wick 2011), the descriptions of fungal communities and of their role in hydrocarbon degradation in marine environments remain scarce. A few reports have shown enrichment of Dothideomycetes-related members, known to be able to degrade PAHs, in oil-polluted beach sediments (Bik *et al.*^2012^) and marshes (Mahmoudi *et al.*^2013^).

### Microbial ecology in chronically PAH-contaminated sites

We consider here chronic pollution corresponding to pyrogenic PAHs, which are considered less bioavailable than petrogenic PAHs (see above sections). They enter the marine environment by deposition on surface waters. Atmospheric PAH depositions have toxic effects on the oceanic phytoplankton communities thus perturbing the first level of marine food webs (Hjorth *et al.*^2007^; Echeveste *et al.*^2010^) and indirectly influencing the other trophic levels, the bacterial and zooplankton communities, through cascading effects (Hjorth, Forbes and Dahllöf 2008). Atmospheric PAHs entering the seawater accumulate in the sea surface microlayer (SML), the ocean/atmosphere interface, with an enrichment factor of up to 500 times in comparison to the concentrations found in the water column (Wurl and Obbard 2004). The role of the biological pump in the sequestration of atmospheric organic pollutants has been described (Galbán-Malagón *et al.*^2012^). This sequestration can be explained by uptake (adsorption and absorption) of organic compounds by phytoplankton, and by fluxes of settling particles rich in organic matter, driving and enhancing air–water diffusive fluxes. The SML is home to a characteristic bacterial community known as Bacterioneuston that has the potential to cope with the presence of contaminants (Sauret *et al.*^2015^). On the French Mediterranean coast, the PAH-degrading bacteria composition was found to be dependent on the PAH source, with the presence of bacterial species different to the well-known PAH degraders and pyrogenic PAHs that were shown to be the most abundant. The PAH degraders in the SML were significantly correlated with the dissolved total PAH concentrations that formed a gradient from the shore to near-shore waters (Sauret *et al.*^2015^).

As described above for the *Deepwater Horizon* oil spill, chronic PAHs were also detected in marine snow (Li *et al.*^2014^), coagulated to small particles and aggregated to organic matter (De La Rocha 2007). As it sinks, marine snow transports PAHs rapidly to the seabed sediments (Berrojalbiz *et al.*^2011^; Nizzetto *et al.*^2012^). In deep-sea sediments in the Arctic Ocean, the PAH concentration decreases with sediment depth and movement from the south to the north (Dong *et al.*^2015^). Using both culture-dependent and independent methods, *Cycloclasticus*, *Pseudomonas*, *Pseudoalteromonas*, *Marinomonas*, *Halomonas* and *Dietzia* have been revealed in the sediment and suggested as major players in PAH degradation under low temperatures (Dong *et al.*^2015^). Populations containing genes encoding the α-subunit of PAH dioxygenase have been described as fairly stable and relatively abundant within the indigenous microbial community in chronically polluted Subantarctic marine sediments (Marcos, Lozada and Dionisi 2009). All these findings reveal that PAHs and PAH-degrading bacteria are widespread in the deep-sea sediments of polar oceans.

### Interactions and environmental parameters affecting microbial communities and PAH biodegradation

Microorganisms constitute community assemblages in natural environments, living in association with other (micro) organisms, ensuring the microbial loop through top-down regulation and ecosystem services by metabolic networks. These interactions, characterized by the coexistence of different species involved in positive and/or negative interactions, are an integral part of the functioning of ecosystems and govern microbial processes involved in the hydrocarbons biodegradation (Head, Jones and Roling 2006; McGenity *et al.*^2012^). Moreover, it is known that in natural or anthropogenic environments some pollutants can only be eliminated through the action of several microorganisms performing additional reactions (Müller 1992; Singleton 1994). Indeed, it is known that natural microbial consortia or networks allow microorganisms to perform complex tasks, including the degradation of recalcitrant molecules such as PAHs (for a review, see McGenity *et al.*^2012^).

### *In situ* characterisation of PAH microbial assemblages

*In situ* studies comparing contaminated and uncontaminated marine sediments have revealed specific microbial assemblages related to the presence of PAHs (Bordenave *et al.*^2008^; Todorova, Mironova and Karamfilov 2014; Wu *et al.*^2014^). The degradation capacities of these microbial assemblages were further attested by the detection of aromatic ring hydroxylating dioxygenase genes (*rhd*), genes involved in the first step of aerobic PAH biodegradation (Bordenave *et al.*^2008^; Todorova, Mironova and Karamfilov 2014; Wu *et al.*^2014^). These genes have been used as target genes for the characterisation of PAH-degrading microorganisms (Ben Said *et al.*^2008^; Guermouche M'rassi *et al.*^2015^) as well as for the estimation of the PAH-degradation potential of microbial communities in the environment (Chadhain *et al.*^2006^; Bacosa and Inoue 2015; Meynet *et al.*^2015^). Interestingly, Xia *et al.* (2015) demonstrated that the diversity of *rhd* genes was correlated with bioavailable PAH contents. The diversity of *rhd* genes was higher in deposited sediments than in suspended sediments and overlaying water (Xia *et al.*^2015^). The role of *rhd* genes in environmental studies has been further demonstrated by targeting their transcripts, which were shown to be induced just after the addition of fresh bioavailable PAHs contained in heavy crude oil (Paissé *et al.*^2012^).

In the absence of functional evidence, the contribution of PAHs to structuring microbial communities could be difficult to evaluate because of the presence of multicontaminants. To overcome these difficulties, the high-throughput sequencing technology associated with appropriate statistical analyses, such as co-occurrence network analyses, offers the possibility of identifying pollutant–degrader interactions (Yergeau *et al.*^2012^; Llado *et al.*^2015^). Applying such an approach, a recent study revealed Actinobacteria ‘specialists’ associated with PAHs and heavy metals (Hg, Cd, Cu, Pb and Zn) in harbour marine sediments (Duran *et al.*2015a). Such an observation highlights the complexity of the interactions prevailing in the microbial assemblages. The microbial collective metabolic diversity copes with the multiple factors that control PAH biodegradation including oxygen and nutrient availability, pH, salinity and the presence of surfactants, co-substrates and multicontaminants (Lu, Zhang and Fang 2011) and facilitates PAH biodegradation (Gallego *et al.*^2014^).

### Experimental ecology for understanding PAH microbial assemblages

Because of the sediment complexity, the environmental observations alone are insufficient to fully understand the mechanisms controlling the microbial assemblages in the presence of PAHs. Laboratory studies, including single-cell physiology as well as appropriate microcosm and mesocosm experiments, are methods that facilitate understanding complex microbial assemblages (Cravo-Laureau and Duran 2014; Röling and Van Bodegom 2014). We relate here the effect of fluctuations in oxygen availability on PAH microbial assemblages and microbial interactions with macroorganisms.

Oxygen and redox oscillations are major fluctuations in estuarine and tidal sediments (Cravo-Laureau and Duran 2014). The effect of such fluctuations on the dynamics of microbial communities and PAH degradation capacities has been demonstrated in several microcosm (Duran *et al.*2015b; Militon *et al.*^2015^) and bioreactor studies (Cravo-Laureau *et al.*^2011^; Vitte *et al.*^2011^, ^2013^). Different PAH-degrading microbial assemblages have been obtained according to the oxygenation regimes (Vitte *et al.*^2011^) with optimal degradation capacities observed either under permanent oxic conditions (Militon *et al.*^2015^) or under anoxic/oxic oscillations (Vitte *et al.*^2011^). However, degradation under oxic/anoxic oscillations was less effective in removing the toxicity, suggesting incomplete PAH mineralisation with the accumulation of toxic metabolites, probably because the microbial assemblage lacked key microorganisms for a complete PAH-degradation metabolic network (Vitte *et al.*^2013^). These observations illustrate the need for better understanding the biogeochemical functioning of polluted environments under fluctuating redox conditions as highlighted by Borch *et al.* (2010).

The influence of PAHs on the interactions between microbial communities and meiofauna has been revealed by microcosms for studies into bioremediation strategies (Louati *et al.*2013a,b; Ben Said *et al.*^2015^). Meio- and macrofauna influence the microbial assemblages, directly as the main predators and indirectly by modifying the environmental conditions. Louati *et al.* (2013b) demonstrated that top-down control by meiofauna was more effective in shaping microbial assemblages than the selective pressure exerted by PAHs. Microbial interactions, including interactions between microbes during hydrocarbon degradation as well as interactions with meio- and macrofauna and plants in oil-polluted marine sediments, have been recently reviewed (McGenity *et al.*^2012^), highlighting the need for a better understanding of microbial interactions, to achieve a more rational approach to the bioremediation of hydrocarbons.

## CONCLUDING REMARKS

Although the issues related to oil pollution, particularly the concerns regarding to PAHs, are increasingly taken into consideration with the introduction of specific countermeasures, PAHs are still threatening environmental health. The marine environment is the major sink and receptacle of PAHs, which enter through different forms whose bioavailability varies according to their origin and their weathering processes. Microorganisms are the main actors in controlling and determining their fate in all marine ecosystems. The biological pump plays a pivotal role in the surface water and water-column trapping of PAHs, originating from both oil spills and atmospheric deposition, and then transporting them to the bottom. The utilisation of this spectacular phenomenon, by promoting it with fertilisers, for cleaning the open oceans and for carbon sequestration is currently under debate (De La Rocha 2007; Nogales *et al.*^2011^). The wide diversity of microorganisms carrying genes involved in both aerobic and anaerobic PAH degradation together, with the presence of specialist degraders, are found in all the ecosystems examined, ensuring the most important depletion route. The degradation activities depend on a complex set of environmental conditions driving the microbial assemblages that microbial ecology ambitions to understand, a challenging objective from both an academic point of view and an applied perspective. Systems biology tools involving meta-omics approaches are promising tools to address the challenge.

The exploitation of oil reservoirs in more and more extreme conditions, such as deep sea and cold areas, in the context of climate change complicates the challenge. The study of oil reservoirs in more extreme conditions is required because these colder environments present different environmental parameters, including hydrostatic pressure, low temperature and water acidification, that may shape microbial processes. Further knowledge of the ecology of the adapted bacterial populations described in deep-sea sediments and of the PAH degraders found in cold Arctic Ocean sediments, both responsible for low PAH-degradation rates, is required to complete the global model of the fate of PAHs in the marine environment.

Additionally, it is of paramount importance to gain information on how the modification of the resulting environmental parameters may affect the microbial assemblages, the biological pump and the global carbon cycle in a climate change scenario.
